# Strain Rate and Anisotropic Microstructure Dependent Mechanical Behaviors of Silkworm Cocoon Shells

**DOI:** 10.1371/journal.pone.0149931

**Published:** 2016-03-03

**Authors:** Jun Xu, Wen Zhang, Xiang Gao, Wanlin Meng, Juan Guan

**Affiliations:** 1 Department of Automotive Engineering, School of Transportation Science and Engineering, Beihang University, Beijing, China; 2 Advanced Vehicle Research Center, Beihang University, Beijing, China; 3 Beijing Key Laboratory for High-efficient Power Transmission and System Control of New Energy Resource Vehicle, Beihang University, Beijing, China; 4 Department of Transportation Engineering, School of Transportation Science and Engineering, Beihang University, Beijing, China; 5 Department of Civil Engineering, School of Transportation Science and Engineering,Beihang University, Beijing, China; 6 School of Material Science and Engineering, Beihang University, Beijing, China; University of Akron, UNITED STATES

## Abstract

Silkworm cocoons are multi-layered composite structures comprised of high strength silk fiber and sericin, and their mechanical properties have been naturally selected to protect pupas during metamorphosis from various types of external attacks. The present study attempts to gain a comprehensive understanding of the mechanical properties of cocoon shell materials from wild silkworm species *Antheraea pernyi* under dynamic loading rates. Five dynamic strain rates from 0.00625 s^-1^ to 12.5 s^-1^ are tested to show the strain rate sensitivity of the cocoon shell material. In the meantime, the anisotropy of the cocoon shell is considered and the cocoon shell specimens are cut along 0°, 45° and 90° orientation to the short axis of cocoons. Typical mechanical properties including Young’s modulus, yield strength, ultimate strength and ultimate strain are extracted and analyzed from the stress-strain curves. Furthermore, the fracture morphologies of the cocoon shell specimens are observed under scanning electron microscopy to help understand the relationship between the mechanical properties and the microstructures of the cocoon material. A discussion on the dynamic strain rate effect on the mechanical properties of cocoon shell material is followed by fitting our experimental results to two previous models, and the effect could be well explained. We also compare natural and dried cocoon materials for the dynamic strain rate effect and interestingly the dried cocoon shells show better overall mechanical properties. This study provides a different perspective on the mechanical properties of cocoon material as a composite material, and provides some insight for bio-inspired engineering materials.

## Introduction

Silkworms and a range of arthropods and insects including spiders and bees have evolved to spin protein fibers that nicely combine high strength and toughness for multiple functions. Natural silk fibers and cocoons are “ideal” defense and survival tools for the animals [1–4], and on the other hand the silk fibers from domesticated *Bombyx mori* silkworms have long been great attractions to the textile industry [5–8].

Cocoons, consisting of twining silk fibers of a continuous length of approximately 1000 m and bonded together by sericin glue, are a typical natural fiber composite with hierarchical structures [1, 9, 10]. The widely studied silk fibers from the *Bombyx mori* silkworm cocoons are used not only as textile materials, but also as multifunctional materials [11, 12]. To realize the biological function of cocoons as a shield for the pupas, the fiber component in cocoons possessing a semicrystalline structure offers balanced stiffness and extensibility, and the binder component sericin which is an amorphous protein helps to maintain the network structure of cocoons.

Up to date, much research focus have been put on the mechanical behaviors of the silk fibers unravelled from cocoons, their structure-property relations and the biodegradability. Some researchers have focused on studying different kinds of cocoons to comprehensively understand the interplay among the multilayer structures of cocoons, the mechanical properties of cocoon fibers, and the “optimized” mechanical properties of cocoons [9, 13, 14]. More recently, cocoons with characteristic mechanical properties resulting from its constituent silk fiber and the corresponding multi-layered structure have been reported to be ideal candidates for bio-inspired engineering design models [9, 15]. Another study showed a strong dependency of the mechanical properties of different cocoon layers on the microstructures regardless of the same composition among these layers through SEM (scanning electron microscopy) images and mechanical testing [16]. The typical mechanical properties, such as Young’s modulus, ultimate strength and thermo-mechanical parameters, were experimentally measured to be different from innermost pelade to the outermost floss in the thickness direction of *Bombyx mori* cocoon. [17]. In particular, an interesting step was made by Blossmen and *et al*. [18] and reported that *Bombyx mori* cocoons were proven to be able to conserve water for the pupa and acting as a humidity trap while offering mechanical protection.

Nevertheless, the most important biological role for cocoon is to protect the pupa from predators’ attacks (e.g. birds’ pecking) which in essence is an engineering problem of impact and dynamic loading events. Previous studies draw major attention on the quasi-static mechanical behavior of the cocoon material while the mechanical behaviors under dynamic conditions with both inertia and strain rate effect need to be evaluated. Mortimer and Drodge et al [19] studied the bullet impact effect on silk fibers and Chen et al [15] reported a quasi-static “impact” experiment and analysis on cocoons. However, a direct measurement of cocoon shells under dynamic loading conditions between quasi-static and impact rates is still lacking. Moreover, more research is required to study the structure-property relationships of the wild cocoon instead of the domesticated ones from a biomimetic point of view. Thus, in the present study, a focused experiment investigation on the mechanical properties of cocoon shells from wild silkworm species *Antheraea pernyi* of three different orientations under various dynamic strain rates is conducted. Furthermore, the microstructural changes of cocoon shells during deformation are observed using scanning electron microscopy to evaluate the effect of microstructure on mechanical behaviors of cocoons. Finally, simple constitutive models are referenced to provide fundamental understanding in the mechanical properties of cocoon shells under dynamic loading conditions.

## Experiment

### 2.1 Materials

The Chinese tussah silkworm *Antheraea pernyi* cocoon we chose for the present study is one of the most common wild cocoons in northern China and no endangered species were involved in this work. All the *Antheraea pernyi* cocoons used in this study were purchased from a professional silk rearing farmer named Desheng Song in Xianrendong Town, Dalian City, Liaoning Province, China. To keep the consistency of all the cocoon samples, they were stored under the same environmental conditions in the laboratory (a temperature below 15°C and low humidity to prevent the live pupas from hatching). In order to study the effect of moisture and the processing of drying which is used in silk industries on the mechanical properties of cocoon shells, approximately half of the overall cocoon shell specimens were dried at a temperature of 120°C in a laboratory oven for 48 hours under full ventilation. Cocoon shell specimens from cocoons as received with live pupas inside serve as a comparison group of the natural untreated ones in material testing.

**Fig 1:**
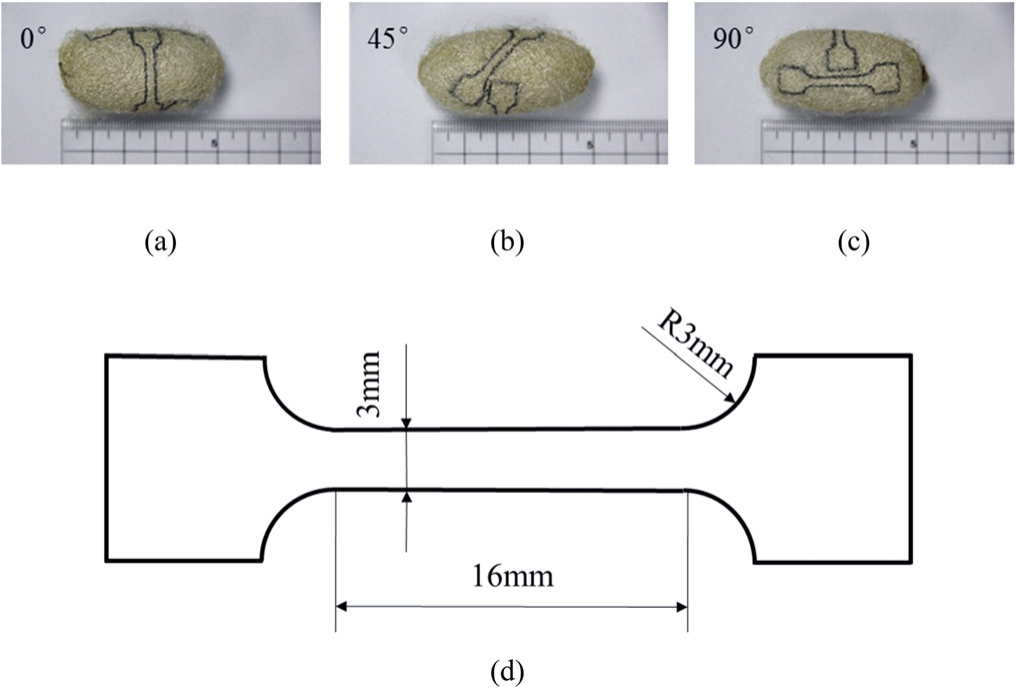
Dog-bone shaped specimens cut from the (a) 0°, (b) 45°, (c) 90° direction of the same contact cocoon and (d) The size of designed dog-bone shaped specimen.

A PD-153 (standard) vernier caliper (Pro’sKit) and a micrometer were used to measure the geometry of the cocoons. For preparing cocoon shell specimens for tensile testing, cocoon shells were first carefully cut open along its long axis to remove the pupas inside to prevent contamination from the body fluid of the pupa. Then as Fig 1 shows, dog-bone shaped specimens were cut from three directions, i.e. 0°, 45°, 90° respectively to the short axis of the cocoon shell and the middle section of the dog-bone specimens were 3 mm in width and 16 mm in length. The thicknesses of the samples were measured through a micrometer, which varied from 0.3 mm to 0.6 mm. Note that the possible delamination of layers and damage were carefully controlled during sample preparation such that its influence on the mechanical behaviors of cocoon material was considered trivial. Considering biological materials are highly variable in both mechanical properties and morphologies, the effect of individual cocoon structural difference is ruled out by comparing only the specimens of three directions from the same cocoons. Each dog-bone shaped specimen was marked and placed in plastic bags before and after the experiment.

### 2.2 Methods

Quasi-static uniaxial tensile tests were performed at room temperature of 14.8°C and relative humidity of 22% on a MTS materials testing machine with a load cell of 0–200 N with a precision grade of 0.5% to accurately record the deformation extension and force of the cocoon specimens. In the meantime, dynamic-rate uniaxial tensile tests were performed utilizing a computer controlled Instron E10000 test machine and the load cell was 0–250 N with a precision of 0.0125 N. The dynamic loadings ranging from 10^−4^ m/s to 0.2 m/s, converted to nominal strain rate of 0.00625 s^-1^ to 12.5 s^-1^, with the consideration of the cocoon anisotropic morphology were tested. The nominal stress and strain was calculated from the load force and cross-head displacement divided by the cross-sectional area and the effective length of the middle section of the dog-bone specimen. The thickness and width were measured at least at two different positions of each dog-bone specimen for its averaged cross-sectional area. Eight repeated tests were conducted for each dynamic rate and each anisotropic specimen. The properties were analyzed and an average and standard deviation for each property was calculated. The fractured specimens were recycled for microstructural observations using a SEM (JSM 6010).

## Results

**Fig 2:**
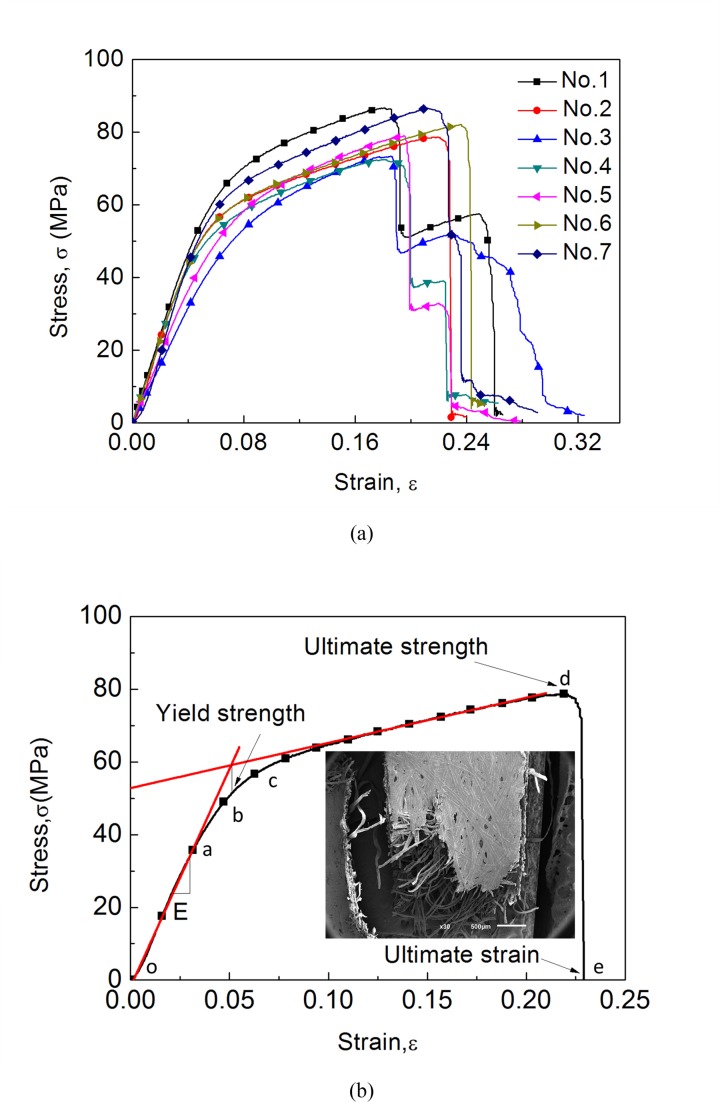
Representative stress-strain curves (a) stress-strain curves of dried dog-bone shaped specimens in the 0-degree direction under quasi-static condition (b) different stages in typical stress-strain curve of dried dog-bone shaped specimen in the 0-degree direction under a nominal strain rate of 0.00625 s^-1^.

The general profiles of mechanical properties of cocoon materials in three different directions are highly reproducible from a number of repeated material tests (*n* = 8). To represent the characteristic stress-strain curve of the cocoon shell specimen without missing the discussion on variability, the representative stress-strain curves are selected to match the averaged properties of each group of stress-strain curves. For example, all the stress-strain curves (representative curve highlighted in red) of dried dog-bone shaped specimens in the 0° direction under quasi-static condition are presented in Fig 2A. Fig 2B illustrates the different stages of the stress-strain behavior of the cocoon shell specimen using a representative stress-strain curve. It appears that the trend of the stress-strain curve of cocoon materials is quite similar to that of steels [20–22], although it is a type of fiber reinforced polymer material, where four stages can be clearly observed, including elastic region (oa), yielding (bc), plastic stage (cd) and fracture (de).

For mechanical property analysis average values and their standard deviation of typical properties, i.e. Young’s modulus *E*, yield strength *σ*_*y*_, ultimate strength *σ*_*u*_ and ultimate tensile strain *ε*_*u*_, are derived from the stress-strain curves and listed in Table 1 (natural dog-bone specimens) and Table 2 (dried dog-bone specimens). Following the trend of the stress-strain curve, firstly, we see the stress linearly increases with strain and the slope decreases gradually at a yield point of approximately 50.9 MPa. Then cocoon shell specimen enters its “plastic” stage, where a post-yield modulus can be obtained. Finally, when stress reaches its maximum value with an ultimate strain higher than 20%, the curve starts to fall rapidly when the cocoon material fails. Evidently, some of the cocoon specimens in the experiment showed progressive and multi-stage failure behavior as shown in Fig 2A. The microstructure of fractured specimen in the insert of Fig 2B suggests that there is no necking but individual fiber break-up along an irregular fracture path through the width of the specimen.

**Table 1:** Average values and standard deviation of typical parameters in stress-strain curves of natural cocoon material.

Nominal strain rate (s^-1^)	Direction	Young’s modulus (GPa)	Yield strength (MPa)	Ultimate strength (MPa)	Ultimate strain
0.00625	0°	0.90±0.17	47.89±3.75	76.08±6.76	0.28±0.02
0.00625	45°	0.86±0.09	46.74±3.24	71.05±1.68	0.27±0.02
0.00625	90°	0.72±0.09	36.60±3.44	54.70±5.85	0.25±0.05
0.0625	0°	1.05±0.16	51.36±5.19	73.83±8.17	0.22±0.05
0.0625	45°	0.86±0.16	46.39±3.48	63.28±6.75	0.21±0.03
0.0625	90°	0.82±0.15	39.17±5.71	55.31±7.16	0.24±0.03
0.625	0°	1.26±0.18	52.68±3.65	79.56±4.63	0.24±0.02
0.625	45°	1.12±0.16	47.43±5.27	67.38±7.82	0.21±0.03
0.625	90°	1.05±0.18	39.64±6.89	57.33±9.24	0.20±0.35
6.25	0°	1.18±0.31	53.77±7.72	78.56±10.38	0.20±0.03
6.25	45°	1.20±0.33	45.59±8.20	62.39±10.69	0.17±0.05
6.25	90°	1.15±0.19	45.39±4.71	63.59±8.50	0.21±0.04
12.5	0°	1.45±0.35	57.37±8.55	85.21±13.43	0.22±0.03
12.5	45°	1.32±0.21	47.24±2.74	64.29±5.58	0.16±0.03
12.5	90°	1.25±0.14	43.07±3.69	60.18±4.46	0.17±0.04

**Table 2:** Average values and standard deviation of typical parameters in stress-strain curves of dried cocoon material.

Nominal strain rate (s^-1^)	Direction	Young’s modulus (GPa)	Yield strength (MPa)	Ultimate strength (MPa)	Ultimate strain
0.00625	0°	1.09±0.17	52.70±4.25	79.38±5.17	0.21±0.02
0.00625	45°	0.99±0.12	45.59±1.82	67.08±5.60	0.18±0.03
0.00625	90°	0.97±0.12	42.28±1.80	59.72±2.10	0.19±0.02
0.0625	0°	1.02±0.16	59.31±5.35	84.92±7.94	0.23±0.02
0.0625	45°	0.95±0.16	51.73±6.82	71.08±9.30	0.20±0.04
0.0625	90°	0.93±0.09	45.01±4.42	61.07±5.98	0.19±0.03
0.625	0°	1.37±0.25	55.63±4.22	79.92±8.58	0.21±0.04
0.625	45°	1.38±0.25	44.53±7.65	63.41±8.67	0.16±0.04
0.625	90°	1.24±0.15	38.43±3.28	55.00±4.23	0.15±0.02
6.25	0°	1.14±0.39	67.48±11.34	92.00±17.63	0.17±0.02
6.25	45°	1.36±0.33	53.19±9.79	74.33±13.96	0.16±0.03
6.25	90°	1.21±0.20	43.73±6.86	61.33±8.52	0.15±0.02
12.5	0°	1.59±0.36	72.44±9.22	86.88±13.19	0.15±0.03
12.5	45°	1.42±0.28	55.75±7.03	73.21±8.77	0.13±0.02
12.5	90°	1.25±0.18	47.18±6.46	62.00±8.04	0.12±0.03

## Discussion

### 4.1 Anisotropy in cocoon shell properties

**Fig 3:**
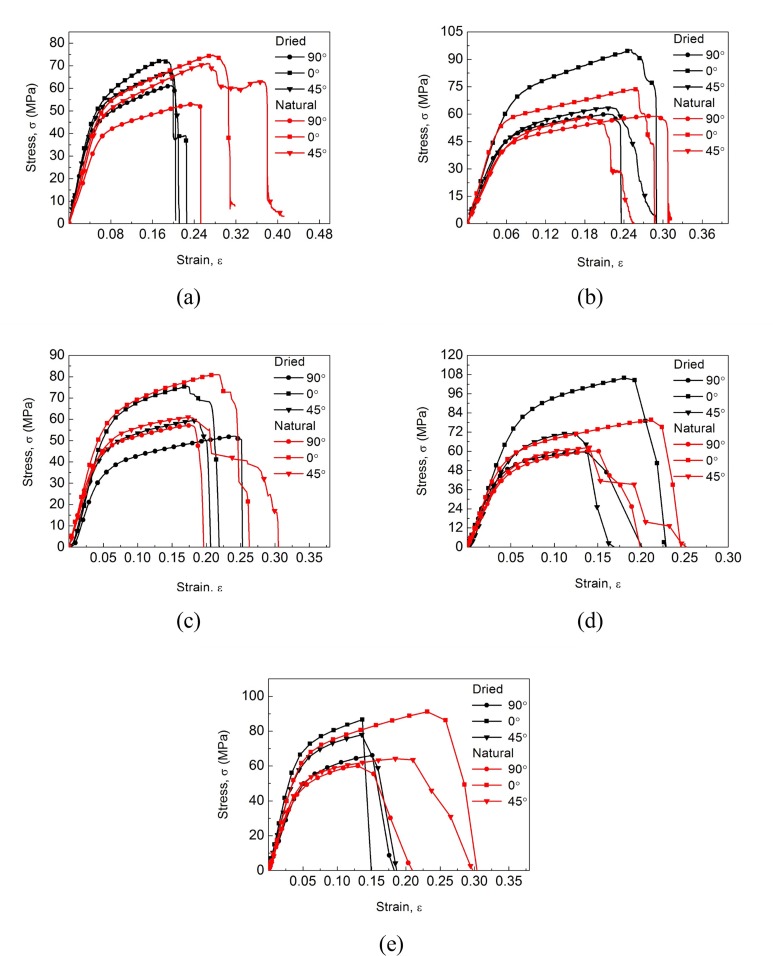
Representative stress-strain curves of dog-bone shaped specimens along different directions with a nominal strain rate of (a) 0.00625 s^-1^ (b) 0.0625 s^-1^ (c) 0.625 s^-1^ (d) 6.25 s^-1^ (e) 12.5 s^-1^.

**Fig 4:**
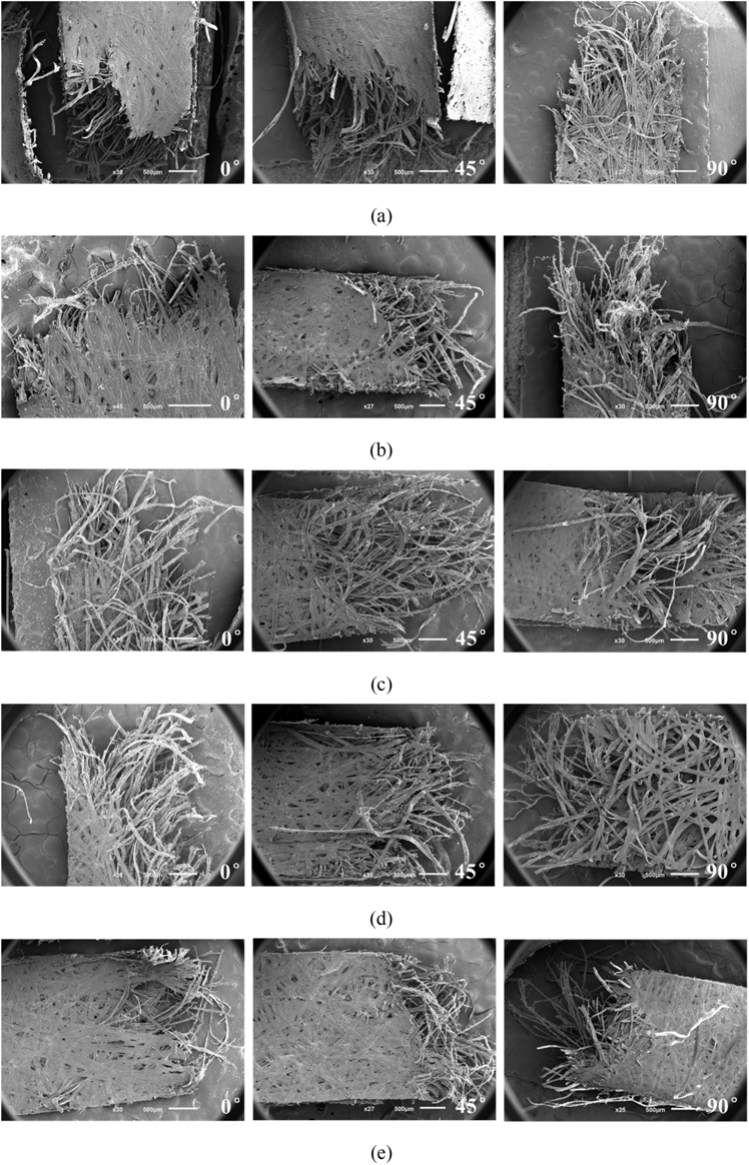
SEM graphs of natural dog-bone shaped specimens along different directions with a nominal strain rate of (a) 0.00625 s^-1^ (b) 0.0625 s^-1^ (c) 0.625 s^-1^ (d) 6.25 s^-1^ (e) 12.5 s^-1^.

Material tensile tests under five different nominal strain rates ranging from 0.00625 s^-1^ to 12.5 s^-1^ were performed for both dried and natural dog-bone shaped specimens along the 0°, 45°, 90° directions from cocoons. Some representative stress-strain curves for each experimental condition and each group of anisotropic specimens are shown in Fig 3. The SEM microstructural images of different strain rate and orientation are inserted for the natural cocoon shell specimens in Fig 4. It is found that all the analyzed mechanical properties including *E* and *σ*_*y*_, *σ*_*u*_ and *ε*_*u*_ are the highest in the 0° direction (along the minor axis of the ellipsoid shaped cocoon), followed by those in 45° direction and samples and in 90° direction. This interesting phenomenon of anisotropic morphology manifestation on the mechanical behaviors of cocoons cannot be accidental, but may have essential meanings for cocoons’ biological functions to protect pupae. As shown in Fig 4, it appears that the fracture of the silk fibers in the specimens in the 0° direction concentrates more along the tensile direction, while along the other two directions fibers tend to distribute in a more random way. Actually, previous studies have shown that the *Antheraea pernyi* silk fiber have powerful mechanical properties with a tensile strength of 0.3–1.0 GPa and a Young’s modulus of approximately 10 GPa, which is about ten times of that of cocoon shells [5, 23–25], indicating that the strength of silk fiber contributes greatly to the strength of the cocoon shell. Therefore, due to the preferential orientation of the silk fibers along the 0° direction there is a tendency that cocoon specimen in the 0° direction show enhanced mechanical properties compared to two other directions. In other words, the preferential orientation of silk fibers in cocoon shells is attributed to the differences in their directional mechanical properties. This designing principle could be used for spatial structures with optimized mechanical integrity given the same limited amount of raw materials based on qualitative speculations in Ref [17]. Another probable explanation is that the 0° direction of the cocoon shell possess a higher density of silk fibers. This may be supported by observations on silkworm’s spinning process, during which the silkworm appears to spin more silk along the short axis circle of the cocoon ellipsoid. This interesting behavior may be driven by the “defense” function of the cocoon and suggests that this particular position of the cocoon may face more attacks than the other places on the cocoon surface. However, further evidence is needed to give conclusive explanation.

Porter and Vollrath [26] put forward a connectivity model to quantitatively describe the stress-strain behaviors of cocoons and other particulate composite materials, where the stress-strain behavior of *Antheraea pernyi* cocoon was also modelled. We found that the anisotropic effect of the cocoon stress-strain behaviors can be well modelled based on the connectivity model, which will be discussed in section 4.3.

### 4.2 The influence of strain rate

**Fig 5:**
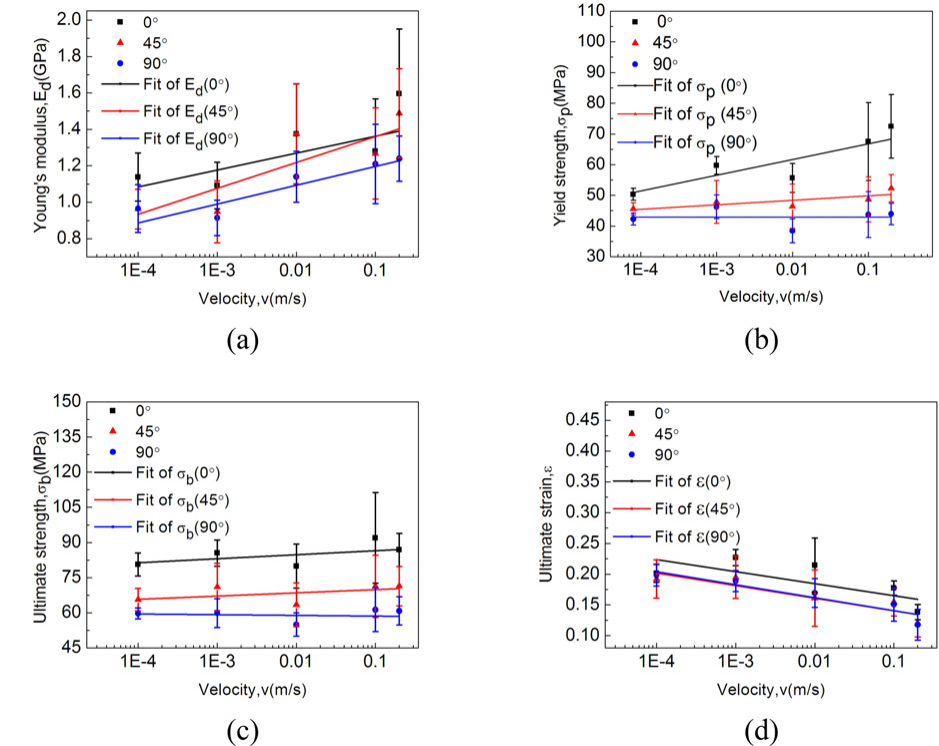
Typical parameters of cocoon materials with the increase of loading rate: the trends of (a) Young’s modulus (b) yield strength (c) ultimate strength and (d) ultimate strain of dried cocoon materials in different directions with increasing loading rate.

The table listed data of averaged typical properties (*E*, *σ*_*y*_, *σ*_*u*_, *ε*_*u*_) of cocoon materials with the increase of nominal strain rate ranging from 0.00625 s^-1^ to 12.5 s^-1^ are also presented and compared in Fig 5. These results show cocoon shell specimens of different orientations have different strain rate sensitivity. Increased strain rate results in significantly increased Young’s modulus, yield strength and decreased ultimate strain for the cocoon specimens along the 0° direction; but for the other two directions, the increased strain rate results in the same trend for Young’s modulus and ultimate strain, but only negligible difference in the yield and ultimate strength.

For polymer materials increasing strain rate has an equivalent effect as decreasing temperature to result in a “hardened” mechanical response [27]. In other words, higher strain rate does not allow polymer chains or segments to relax, hence a higher modulus is often observed accompanying the higher strain rate. The decrease in the ultimate strain can also be explained by the lack of polymer molecular relaxation due to the increased strain rate and shorter response times. Drodge and et al. have studied *high* strain-rate constitutive behavior of *Bombyx mori* silk fibers[19]. Other semi-crystalline polymers have been reported to have strain-rate sensitive (dynamic rate range) stress-strain behaviors [28, 29], although this effect on silkworm cocoons was not studied in detail. Coming also from metal materials background, we see cocoons behave similar to other strain rate dependent materials including high strength steels [21, 22, 30], whose yield stress and ultimate strength tend to increase to keep pace with the increasing strain rate. Bearing in mind that the strain-rate dependence of cocoon shells could be intrinsically determined by the strain-rate dependence of the constituent silk fibers and binder, we propose to understand the dynamic strain rate dependence of cocoon shells using well-established models for structural materials and fiber composites.

### 4.3 Modelling the anisotropy and strain-rate dependence of mechanical properties of cocoon shells

Chen *et al*.[26] constructed a connectivity model (strain-activated), which is widely used for predicting non-woven composite properties, for understanding the cocoon shell stress-strain mechanical properties using a typical fiber-binder bonded morphology. Eq (1) shows a modulus or stiffness decreases as the numbers of broken bonds are activated by increasing strain which can be quantified by an Arrhenius activation function.

$$\sigma =Y\varepsilon \left\{1-\sum_{i}f_{i}\;\text{exp}\left[-{\left(\frac{\varepsilon_{ai}}{\varepsilon }\right)}^{2}\right]\right\}$$ (1)

where *f*_*i*_ = *Y*_*i*_ / *Y*, *Y* is the initial elastic modulus and *ε*_*a*_ is the activation strain according to different bonding/connectivity mechanism.

In view of the obvious strain rate dependency of the observed tensile experimental results, a modification of Eq (1) to describe the dependency of both static and dynamic strain rates of cocoon materials is showed in Eq (2).

$$\sigma =Y\varepsilon \left\{1-f\;\text{exp}\left[-{\left(\frac{\varepsilon_{a}}{\varepsilon }\right)}^{2}\right]\right\}(1+D\;\text{ln}\frac{\dot{\varepsilon }}{{\dot{\varepsilon }}_{0}})$$ (2)

where *D* is a parameter showing strain rate sensitivity of cocoon materials. Here we use only one strain activation mechanism (e.g. bonds lost through the yield of sericin binder), and the strain rate dependency part on the right in Eq (2) is adapted from the Johnson-cook model which will be discussed next. The model parameters of *f*, *ε*_*a*_ as shown in Table 3 are derived from the experimental data at quasi-static rate and then applied to predict the mechanical properties of cocoon materials at dynamic strain rates. The 90^o^ direction for both dried and natural cocoons gives a greater “*f*” and a slightly smaller “*ε*_*a*_”, which suggests that the sericin bonding connectivity in the 90^o^ direction contributes more to the overall stress-strain behavior. “*D*” shows a clear trend of decreasing with the increase of the orientation degrees for the dried cocoons, which suggests that the 0^o^ direction has a greater strain-rate sensitivity.

**Table 3:** Parameters of the improved constitutive models.

Parameters	*f*	*ε*_*a*_	*D*
dried	0°	0.6381	0.0956
dried	45°	0.7332	0.0595
dried	90°	0.7494	0.0750
natural	0°	0.7554	0.0891
natural	45°	0.7508	0.0855
natural	90°	0.7567	0.0847

**Fig 6:**
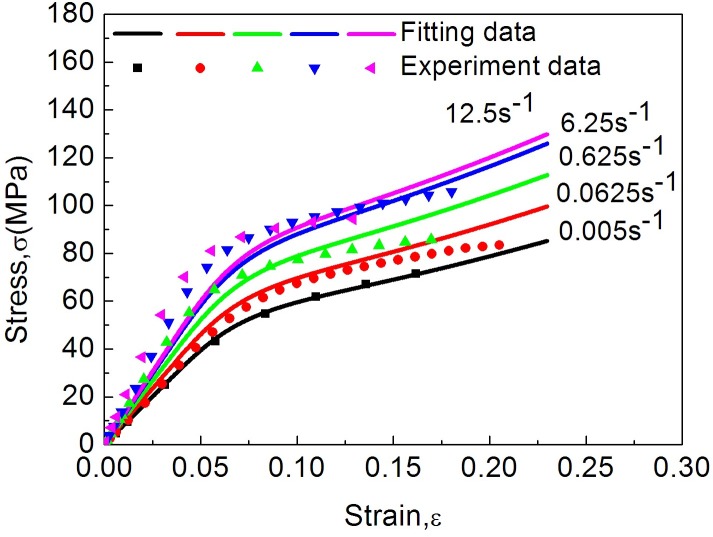
Comparison between experimental and the improved model under different strain rates.

Fig 6 shows the modelled and experimental stress-strain curves of the dried cocoon shell specimens, which suggests a good agreement between the experiments and the improved model and can be used for predicting the stress-strain behaviors of cocoon shells under varied dynamic loading rates. Nevertheless, at higher strain rate, e.g. 12.5 s^-1^, the model curves start to deviate from the experimental curves which may require a further understanding of the combinational effect of the strain rate on both the non-woven structure and the material properties of cocoons.

Johnson-Cook model is one of the most widely used constitutive models to describe strain rate dependency in metals [31–35] and it is interesting to find out the tensile behavior of cocoon materials has similar profiles as metals under quasi-static and dynamic strain rates. Although materials properties and structural changing mechanisms are distinct between metals and silk, the form of Johnson-Cook model could be used to mathematically describe the strain rate dependence of mechanical properties of cocoon materials. The expression of the Johnson-cook model is written as

$$\sigma =(A+B\varepsilon_{p}{}^{n})(1+C\;\text{ln}\frac{\dot{\varepsilon }}{{\dot{\varepsilon }}_{0}})$$ (3)

where *σ* is the stress, *ε*_*p*_ is the plastic strain and $\dot{\varepsilon }/{\dot{\varepsilon }}_{0}$ is dimensionless relative strain rate, with ${\dot{\varepsilon }}_{0}=0.005\;{\text{s}}^{-1}$ as a reference strain rate, *A*, *B*, *C* and exponential term *n* are four material parameters. Note that this model is used for predicting the post-yield plastic mechanical behaviors and the strain rate dependence of metals. *A* is the yield stress of cocoon materials at a strain rate of 0.005 s^-1^, *B* and *n* reflect the nonlinearity of stress-strain behaviors of the cocoon materials after yield, which can be obtained by curve fitting at a strain rate of 0.005 s^-1^, while *C* is a parameter showing strain rate sensitivity of cocoon materials, determined by plotting different yield stresses against different relative strain rates and calculating the slope. Table 4 summarized parameters of this model which could describe the post-yield mechanical behaviors of cocoon materials of three orientations. The values of the strain-rate parameter *C* are directly used as *D* in the improved connectivity model.

**Table 4:** Parameters of the fitted strain rate dependent constitutive models.

Parameters		A (MPa)	B (MPa)	n	C
dried	0°	50.16	110.11	0.687	0.067
dried	45°	45.59	65.46	0.060	0.013
dried	90°	42.28	58.26	0.585	0.002
natural	0°	48.13	72.32	0.647	0.042
natural	45°	45.01	77.74	0.687	0.024
natural	90°	37.59	73.86	0.228	0.028

**Fig 7:**
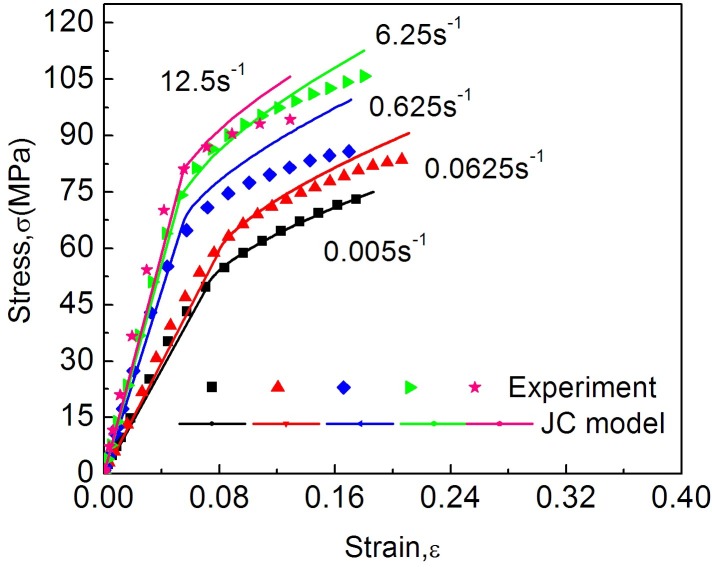
Comparison between experimental and the Johnson-Cook model under different strain rates.

The comparison between experimental and the Johnson-Cook model is presented in Fig 7. Take 0° direction of the dried cocoon materials for example. The Johnson-Cook model could nicely reflect the trend of modulus decreasing above the yield for cocoon shell structures.

To summarize the section, a modified connectivity model with the introduction of the strain-rate dependence parameter could well describe the overall stress-strain behaviors and the strain rate dependency of the cocoon shell materials, and the strain-rate sensitivity of cocoon shell materials may use the mathematical description from Johnson-cook model, which well-explains the post-yield tensile mechanical behaviors of cocoon shells.

### 4.4 The differences between dried and natural cocoon materials

**Fig 8:**
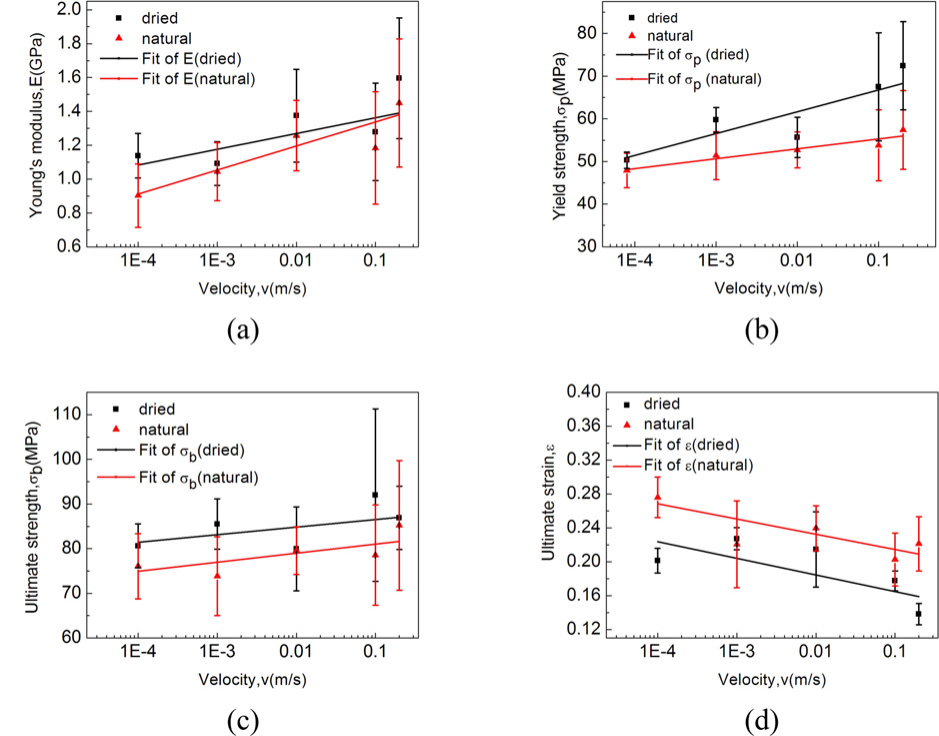
Comparison of some typical parameters ((a) Young’s modulus (b) yield strength (c) ultimate strength (d) ultimate strain) between dried cocoon materials and natural cocoon materials.

The general variation trends of the main mechanical property indexes of both dried and natural cocoon materials with the increase of loading rate are shown in Fig 8. There is a tendency that *E*, *σ*_*y*_ and *σ*_*u*_ of dried cocoon materials are higher than those of natural ones. In addition to that, dried cocoon materials appear to have lower ultimate strain when compared with natural cocoon materials as well. In other words, natural cocoons with water lubrication tend to possess lower Young’s modulus but higher ultimate strain as a trade-off which may result in an overall larger deformation before the cocoon fails. Previous study [36] has shown that silk fibers from natural cocoons becomes stiffer after being heated at the temperature of 120°C. Furthermore, since silkworms spin protein polymers into multifunctional silk fiber as an essential building block of cocoons, cocoon materials dried at a temperature of 120°C in a laboratory oven for 48 hours under full ventilation are expected to have changed physicochemical properties and protein structures of silks due to the loss of water molecules, resulting in enhanced mechanical properties such as higher modulus and strength in an engineering sense.

## Concluding Remarks

Cocoons that naturally evolve to act as impact-resistant armor for silkworm species may serve as an inspiration for impact protection material designs in engineering applications. In this paper, we focused on the anisotropic shape effect and the dynamic-rate effect on tensile mechanical properties of the cocoon shell from silkworm species *Antheraea pernyi*. Mechanical properties including Young’s modulus, yield strength, ultimate strength and ultimate strain of the cocoon shell material have been shown to depend on the microstructure of the cocoon ellipsoid, which may be further attributed to the silk fiber orientation relative to the short axis of the cocoon ellipsoid. It is interesting that the cocoon material specimens in the cocoon short-axis direction showed enhanced mechanical behaviors compared to the specimens in the other directions. Furthermore, we investigated the strain rate dependence of the tensile mechanical behaviors of the cocoon shell material. Young’s modulus and ultimate strain showed more obvious sensitivity to strain rate, and higher dynamic rates resulted in significantly greater modulus and suppressed strain. Connectivity model and Johnson-cook model were referenced and modified to describe the mechanical properties of the cocoon materials upon the strain rate dependency. Additionally, the comparison between dried and natural cocoon materials showed that dried cocoon materials possessed enhanced mechanical properties, which suggests thermal treatments through the removal of water and the structural changes in the silk fibers may be used to tailor the overall mechanical performances of the cocoon materials.

This research serves as a preliminary step toward understanding the strain rate dependent mechanical behaviors of cocoons and other natural materials, and hence may inspire us to understand the mechanisms of biological defense and further to facilitate the design of impact-resistant artificial composites in the future.
